# *Tal2* expression is induced by all-*trans* retinoic acid in P19 cells prior to acquisition of neural fate

**DOI:** 10.1038/srep04935

**Published:** 2014-05-12

**Authors:** Takanobu Kobayashi, Rie Komori, Kiyoshi Ishida, Katsuhito Kino, Sei-ichi Tanuma, Hiroshi Miyazawa

**Affiliations:** 1 Kagawa School of Pharmaceutical Sciences, Tokushima Bunri University, 1314-1 Shido, Sanuki, Kagawa 769-2193, Japan; 2 Department of Biochemistry, Faculty of Pharmaceutical Sciences, Tokyo University of Science, 2641 Yamazaki, Noda, Chiba 278-8510, Japan

## Abstract

TAL2 is a member of the basic helix-loop-helix family and is essential for the normal development of the mouse brain. However, the function of TAL2 during brain development is unclear. P19 cells are pluripotent mouse embryonal carcinoma cells that adopt neural fates upon exposure to all-*trans* retinoic acid (atRA) and culture in suspension. We found that the expression of *Tal2* gene was induced in P19 cells after addition of atRA in suspension culture. *Tal2* expression was detected within 3 h after the induction, and had nearly returned to basal levels by 24 h. When GFP-tagged TAL2 (GFP-TAL2) was expressed in P19 cells, we observed GFP-TAL2 in the nucleus. Moreover, we showed that atRA and retinoic acid receptor α regulated *Tal2* expression. These results demonstrate for the first time that atRA induces *Tal2* expression in P19 cells, and suggest that TAL2 commits to the acquisition of neural fate in brain development.

TAL2 is a transcription factor belonging to the basic helix-loop-helix (bHLH) family^1^,^2^. The *Tal2* gene is transcribed in SUP-T3 cells derived from a human T-cell leukemia; these cells harbor the t(7;9)(q34;q32) chromosomal translocation^3^. A translocation between chromosomes 7 and 9 is associated with human T-cell acute lymphoblastic leukemia (T-ALL)^4^,^5^. Thus, TAL2 may be an oncogene that is activated as a consequence of the chromosomal translocation in T-ALL^6^. Moreover, *Tal2* overexpression is confirmed in some lung adenocarcinoma tumor tissues^7^ and conversely, *Tal2* expression is significantly lower in ovarian carcinoma tissue than that in normal tissues^8^,^9^.

Although aberrant TAL2 expression may be involved in development of some cancers, TAL2 is essential for the normal development of the mouse brain. *Tal2* is expressed during embryogenesis, and sites of *Tal2* expression include specific regions of the diencephalon, mesencephalon and metencephalon^10^. *Tal2*-null mutant mice are viable at birth and initially appear normal. However, they develop signs of runting and die between 13 and 32 days after birth; additionally, they display a prominent hydrocephalus at death^11^. These findings indicate that TAL2 plays a pivotal role in development of the mature central nervous system; nevertheless the function of TAL2 in the development processes remains unclear.

P19 cells are a line of pluripotent mouse embryonal carcinoma (EC) cells derived from a teratocarcinoma that formed following transplantation of a 7.5 day embryo into a testis^12^. P19 cells can be induced to differentiate into derivatives of three germ layers–endoderm, mesoderm, or ectoderm–depending on the inducers and the culture conditions. Two different treatments–addition of all-*trans* retinoic acid (atRA) and suspension culture for cell aggregation–are required to induce neural differentiation of P19 cells^12^,^13^,^14^. Various genes–including *Neurod*, *Neurog1*^15^ and *Fgf8*^16^–that are implicated in neural development are induced in P19 cells following these treatments and during the subsequent neural differentiation. atRA is a biologically active form of vitamin A that functions as the activating ligand for three isotypes of retinoic acid receptor (RAR); these isotypes–α, β, and γ–can each form heterodimers with retinoid X receptors (RXRs)^17^. These receptors transduce atRA signals, belong to the family of nuclear receptors, and induce the transcription of target genes when bound with atRA^18^,^19^. atRA plays an important role in early embryonic development and in differentiation of the nervous system including patterning of the brain, specification of neural cell fate, and stimulation of neurite outgrowth^20^,^21^. Additionally, atRA can induce EC cells, including P19 cells, and embryonic stem cells to adopt neural fates^22^,^23^. P19 cells have been used as model cells for the studies of neural differentiation, and these studies have led to the discovery of a number of genes that are important for neural development *in vivo*^12^,^24^,^25^. To understand how the neural fate is acquired in early embryonic development, we have attempted to identify genes that commit to the neural fate using P19 cells that were introduced into the neural differentiation. In these studies, we found that *Tal2* expression was immediately upregulated in P19 cells. Here, we investigated the relationship of atRA, cell aggregation, and *Tal2* induction in P19 cells.

## Results

### *Tal2* was transiently expressed in P19 cells introduced into the neural differentiation

**Figure 1:**
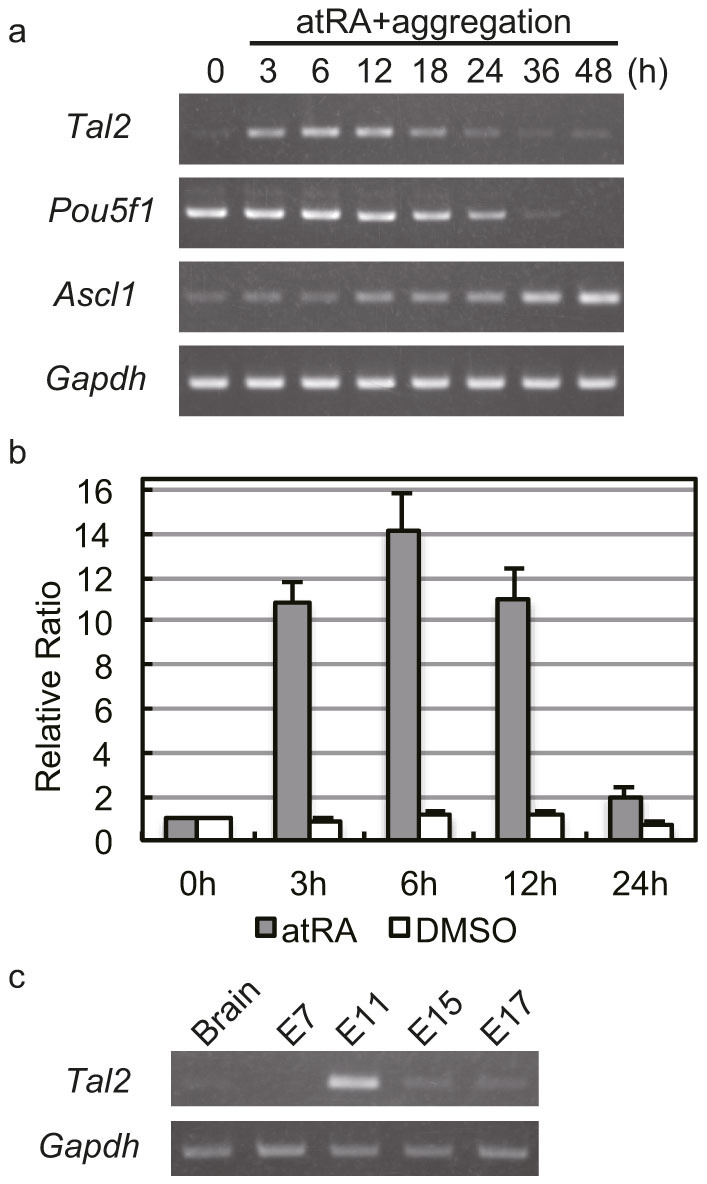
*Tal2* was transiently expressed in P19 cells introduced into the neural differentiation and in embryogenesis. P19 cells were treated with 1 μM atRA in suspension culture. (a) RT-PCR was performed to examine *Tal2*, *Pou5f1* (the pluripotent transcription factor), *Ascl1* (the neurogenic bHLH transcription factor), and *Gapdh* expression in P19 cells at 0, 3, 6, 12, 18, 24, 36 and 48 h. These images were cropped. Full-length gels are presented in Supplementary Figure S2. (b) Real-time PCR was performed to measure *Tal2* expression at 0, 3, 6, 12 and 24 h. The gene expression levels of *Tal2* were normalized using *Hmbs* gene as an internal standard. Fold expression was defined as fold increase relative to 0 h. Data represent the mean ± S.E. of three independent experiments. (c) RT-PCR was performed to examine *Tal2* expression in mouse embryogenesis (E7, E11, E15 and E17) and adult brain. *Tal2* expression was evident at E11. These images were cropped. Full-length gels are presented in Supplementary Figure S3.

We found that *Tal2* expression was upregulated in P19 cells introduced into the neural differentiation for our study using DNA microarray experiments (unpublished data). To study the alteration of *Tal2* expression in more detail here, we extracted RNA from P19 cells 0, 3, 6, 12, 18, 24, 36 and 48 h after addition of atRA in suspension culture, and investigated *Tal2* expression in P19 cells (Fig. 1a). We also examined the expression of *Pou5f1* gene (also known as *Oct3/4*), which encodes the transcription factor in pluripotent cells, and *Ascl1* gene (also known as *Mash1*), which encodes a neurogenic bHLH transcription factor, to assess whether P19 cells were induced to neural cell fate. *Pou5f1* expression decreases during neural differentiation of P19 cells^26^,^27^; contemporaneously, *Ascl1* expression increases^27^,^28^. As previously reported, *Pou5f1* expression decreased and *Ascl1* expression increased contemporaneously in atRA-treated, aggregated P19 cells. This observation demonstrated that P19 cells lost the pluripotency by addition of atRA in suspension culture and were entered into neural cell fate. *Tal2* expression increased within 3 h after treatment, and *Tal2* mRNA levels was returned to the basal levels within 24 h after treatment. In contrast, the expression of all three genes–*Tal2*, *Pou5f1*, and *Ascl1*–was not altered by DMSO treatment in suspension culture (see Supplementary Fig. S1 online).

Next, we used real-time PCR assays to measure *Tal2* expression in P19 cells (Fig. 1b). *Tal2* expression increased 3 h and 6 h after treatment. A reduction in *Tal2* expression from peak levels was first observed at 12 h and *Tal2* expression was close to the basal levels at 24 h. These results indicate that *Tal2* expression is altered in P19 cells for one day.

It has been reported that *Tal2* expression was observed in embryonic head (E10.5 to E14.5)^10^. We also confirmed *Tal2* expression in embryogenesis (Fig. 1c). For this purpose, we used a mouse cDNA panel and RT-PCR. As previously reported, *Tal2* expression was evident at E11 and was not observed in adult brain. In addition, *Tal2* was also hardly observed in E7, E15 and E17. The transient expression of *Tal2* was confirmed in mouse embryogenesis, as well as *Tal2* transiently expressed in P19 cells by addition of atRA in suspension culture.

### Localization of TAL2 in P19 cells

**Figure 2:**
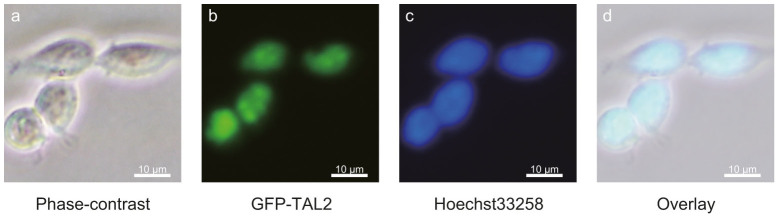
The localization of GFP-TAL2 visualized by fluorescence microscopy. P19 cells were transfected with the expression vector encoding GFP-TAL2 and were stained with Hoechst33258 48 h after transfection. (a) Phase-contrast. (b) GFP-TAL2. (c) Hoechst33258. (d) Overlay.

To examine the behavior of TAL2 in P19 cells, we generated GFP-tagged TAL2 (GFP-TAL2) expression construct. This construct was introduced into P19 cells via transfection, and these cells were stained with Hoechst33258 48 h post transfection. We then used fluorescence microscopy to visualize these cells (Fig. 2). GFP-TAL2 expressed in P19 cells was detected in nuclei.

### atRA induced the expression of *Tal2* gene in P19 cells

**Figure 3:**
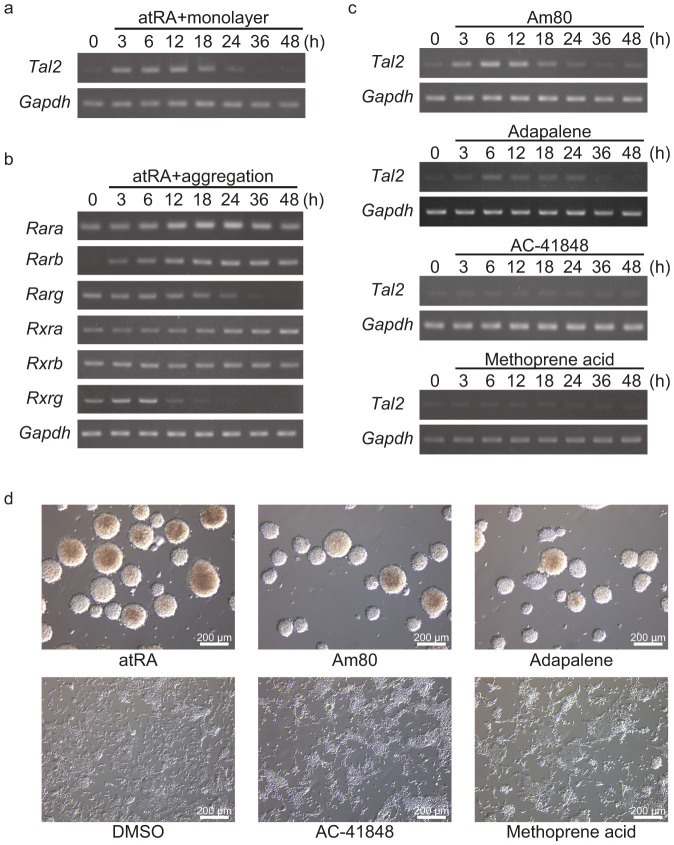
atRA were involeved in *Tal2* expression in P19 cells. (a) P19 cells were treated with 1 μM atRA and cultured in adherent conditions for 0, 3, 6, 12, 18, 24, 36 and 48 h. RT-PCR was performed to examine *Tal2* expression in these cells. *Tal2* expression was induced by atRA treatment of cells in adherent conditions as well as those in suspension culture. These images were cropped. Full-length gels are presented in Supplementary Figure S4. (b) The expression of *Rar* genes and *Rxr* genes in P19 cells after addition of atRA in suspension culture. These images were cropped. Full-length gels are presented in Supplementary Figure S5. (c) RT-PCR was performed to examine *Tal2* expression in P19 cells that were treated with agonists in suspension culture–Am80 (RARα agonist), adapalene (RARβ and RARγ agonist), AC-41848 (RARγ agonist) or methoprene acid (RXR pan-agonist). These images were cropped. Full-length gels are presented in Supplementary Figure S6. (d) Morphology of P19 cells following treatment with atRA, DMSO, or each or four agonists for 48 h.

atRA and cell aggregation are important for neural differentiation of P19 cells^12^,^13^,^14^. Thus, we examined how these treatments were involved in the expression of *Tal2* gene. When P19 cells were treated with atRA and cultured as a monolayer in culture dishes, *Tal2* expression was nearly the same as that in atRA-treated and cell-aggregated P19 cells (Fig. 3a). Additionally, the *Tal2* gene was not induced by DMSO treatment in suspension culture of P19 cells (see Supplementary Fig. S1 online). These results indicate that the induction of *Tal2* in P19 cells is associated with atRA.

atRA binds to the RARs (RARα, β, or γ), and RARs form heterodimers with RXRs (RXRα, β, or γ). atRA-bound RAR/RXR heterodimer can regulate the transcription of target genes. To elucidate the involvement of these receptors in *Tal2* induction, we confirmed the expression of *Rar* genes and *Rxr* genes in P19 cells after addition of atRA in suspension culture (Fig. 3b). For these six genes, the expression of *Rara*, *Rxra* or *Rxrb* was not altered at 48 h. The expression of *Rarg* and *Rxrg* had decreased relative to basal levels. On the other hand, *Rarb* expression was not evident at 0 h, but it was evident within 3 h after treatment, as it has been reported that *Rarb* was induced by retinoic acid in P19 cells^29^,^30^. The induction of *Tal2* and *Rarb* were similar in P19 cells, but *Rarb* expression was not altered from 3 h to 48 h while the expression of *Tal2* gene had decreased from peak levels by 24 h.

Next, in order to investigate the relation between atRA and its receptors, we used four agonists instead of atRA; Am80^31^ (RARα agonist), adapalene^32^ (RARβ and RARγ agonist), AC-41848 (RARγ agonist) and methoprene acid^33^ (RXR pan-agonist). Am80 was able to induce *Tal2* gene as well as atRA. Adapalene also induced *Tal2* in P19 cells, but the induction of *Tal2* by adapalene was weaker than that by atRA or Am80. In contrast, neither AC-41848 nor methoprene acid induced *Tal2* expression (Fig. 3c). Moreover, We also assessed morphological changes of P19 cells that were treated with atRA, DMSO, or each agonist (Fig. 3d). P19 cells formed spherical aggregates called embryoid bodies in the presence of atRA, Am80 or adapalene. Whereas, DMSO, AC-41848 or methoprene acid did not formed embryoid bodies.

### *Tal2* expression was induced by RARα

Adapalene formed P19 cells into embryoid bodies, and weakly induced the expression of *Tal2* compared with atRA or Am80. Moreover, it has been reported that adapalene has stimulated RARα weakly^32^. Although the expression of *Rarb* gene was not observed at 0 h in P19 cells, RARα protein was expressed at 0 h. Based on these findings, we assumed that RARα had an intimate involvement in *Tal2* induction in P19 cells.

**Figure 4:**
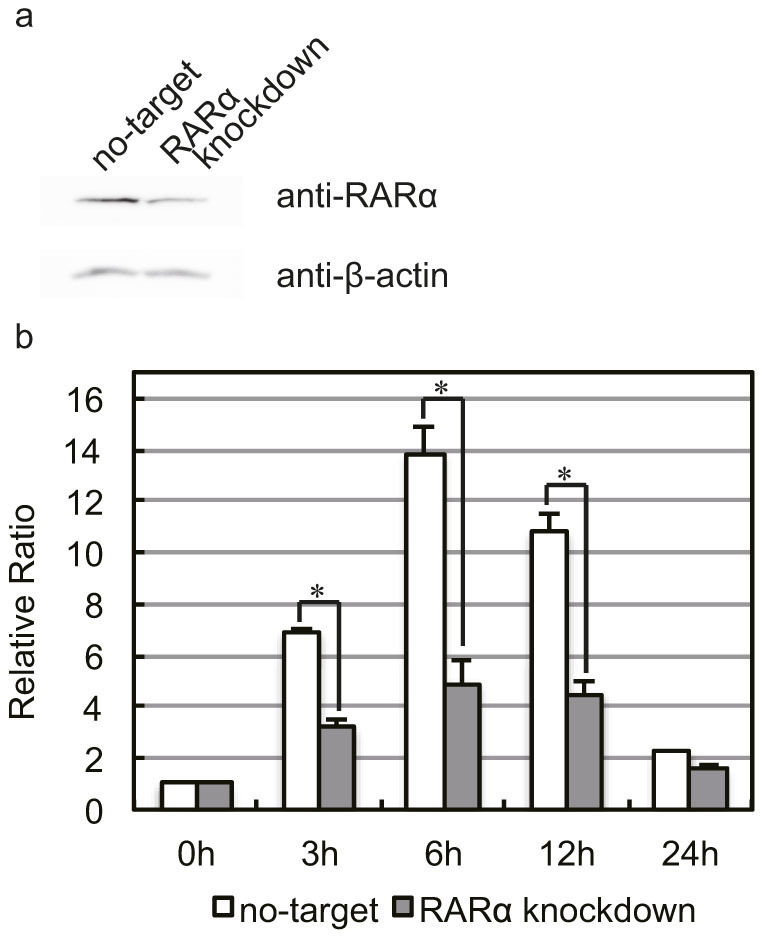
*Tal2* expression was suppressed by RARα knockdown. (a) The suppression of RARα in P19 cells was verified by Western blotting. The protein expression levels were normalized using β-actin as an internal standard, and RARα expression was examined under the same conditions as β-actin. These images were cropped. Full-length gels are presented in Supplementary Figure S7. (b) Real-time PCR was performed to measure *Tal2* expression in P19 cells subjected to RNAi-mediated RARα knockdown. A significant reduction of *Tal2* expression was observed following RARα knockdown. Data represent the mean ± S.E. of three independent experiments. *, *p* < 0.05.

We used RNA interference to suppress RARα expression in P19 cells, and examine the involvement of RARα and the induction of *Tal2*. The RNAi-mediated suppression of RARα in P19 cells was verified by Western blotting (Fig. 4a). *Tal2* expression significantly decreased by RARα suppression in P19 cells after addition of atRA in suspension culture (Fig. 4b). These results indicate that atRA-RARα complexes induce *Tal2* gene in P19 cells.

## Discussion

P19 EC cells are induced into neural differentiation by addition of atRA in suspension culture^13^,^14^. We explore the molecular mechanisms of the entry into neural differentiation using P19 cells. We found that *Tal2* expression was altered rapidly and transiently in these cells (Fig. 1a and b). Interestingly, the transient expression of *Tal2* gene was observed at the stage of organogenesis during embryonic development (Fig. 1c). *Tal2* expression in embryonic head was reported previously^10^,^11^, and TAL2 is inferred to play a role at the early stage of brain development. Because P19 cells have been used as model cells for the studies of neural differentiation, a detailed investigation of the molecular mechanisms regulating *Tal2* expression in P19 cells may be key to understanding the function of TAL2 in brain development.

We investigated the correlation of atRA, cell aggregation and *Tal2* induction in P19 cells. As a result, atRA participated in *Tal2* induction in P19 cells, but cell aggregation was not necessary for *Tal2* induction (Fig. 3a). Next, we examined the involvement of RAR/RXR in the induction of *Tal2* in P19 cells (Fig. 3c). Am80, a RARα agonist, was able to induce *Tal2* expression in P19 cells. Adapalene also weakly induced *Tal2* in P19 cells. Although adapalene is an agonist of RARβ and RARγ, it might also react to RARα in P19 cells. Indeed, RARα is less responsive to adapalene than RARβ and RARγ, and RXRα is no responsive to adapalene^32^. *Rarb* gene was not expressed in P19 cells at 0 h, and was induced by atRA as well as *Tal2* gene. Therefore, adapalene is thought to stimulate the RARα signaling weakly and to induce the expression of *Tal2* in P19 cells. Moreover, RARα knock down by RNA interference suppressed *Tal2* expression in P19 cells (Fig. 4b). These results indicate that atRA-RARα complexes induce transcription of *Tal2* gene in P19 cells.

The embryoid bodies were formed by the agonists, atRA, Am80 and adapalene, that induced the expression of *Tal2* in P19 cells (Fig. 3d). These results raise the possibility that TAL2 is also involved in the formation of embryoid bodies of P19 cells. TAL2 induced by these agonists might activate genes such as *Fgf8*^34^ as a transcription factor that are involved in cell aggregation.

The bHLH family to which TAL2 belongs regulates a variety of biological processes. The bHLH usually has two functionally distinct domains–the basic domain and the HLH domain. The bHLH proteins form dimers via HLH domains and bind DNA via the basic domains. These dimers activate target genes in the nucleus^2^. Therefore, TAL2, which is a bHLH protein, is probably involved in transcriptional activation of target genes in the nucleus. Indeed, the GFP-TAL2 fusion protein that we generated localized to nuclei (Fig. 2). Moreover, GFP-TAL2 was highly enriched in a part of each nucleus. In zebrafish, TAL2 is expressed in the lateral floor plate of the spinal cord^35^. The floor plate is an important signal center that specifies neurons and glia cells in the ventral neural tube and guides the trajectory of outgrowing axons. TAL2 acts upstream of the *gad67* gene that is the GABA-synthesizing enzyme glutamic acid decarboxylase in Kolmer-Agduhr″ (KA″) cells of the lateral floor plate^36^. However, it is not clear whether the mammalian spinal cord has KA″ cells, and *Tal2* has not yet been observed in the mouse spinal cord.

atRA is important for embryogenesis and the differentiation of the nervous system, and we demonstrated for the first time that *Tal2* expression was induced by atRA, which introduced P19 cells into neural differentiation. In addition, the transient expression of *Tal2* was observed in organogenesis of development. These findings suggest that TAL2 plays a role in acquisition of neural cell fate as a transcription factor in neural differentiation. Because the neural differentiation of P19 cells in part mimics the development of the nervous system, P19 cells are useful for studying the molecular mechanism of TAL2 in neural differentiation. Further work is underway to clarify the function of TAL2 in neural differentiation using the differentiation system of P19 cells.

## Methods

### Cell culture and neural differentiation

P19C6, a subclone of P19 mouse embryonic carcinoma cell line, was used in this study^37^. P19C6 was provided by the RIKEN BRC through the National Bio-Resource Project of the MEXT, Japan. P19 cells were cultured in α-MEM (Sigma-Aldrich, MO) supplemented with 10% FBS (Life Technologies, CA) and 2 mM L-glutamine (Kanto Chemical, Japan). To induce neural differentitation, these cells were aggregated in a suspension culture dish (SUMILON, Japan) at a seeding density of 2 × 10^5^ cell/ml in the presence of 1 μM atRA (Sigma-Aldrich), which was dissolved in DMSO (Sigma-Aldrich)^28^. DMSO concentration in culture condition was 0.01%. To identify the RAR and RXR subtypes, 100 nM Am80 (RARα agonist), 100 nM adapalene (RARβ and RARγ agonist), 100 nM AC-41848 (RARγ agonist), or 100 nM methoprene acid (RXR pan-agonist) were used in place of atRA^28^,^29^. These agonists were purchased from Sigma-Aldrich.

### RT-PCR and real-time PCR

RNeasy Mini Kit (QIAGEN, Germany) was used accoding to the manufacturer's instructions to isolate total RNA from P19 cells. RT-PCR was performed as described previously^27^. In brief, reverse transcription was performed with 1 μg of total RNA and SuperScript III reverse transcriptase (Life Technologies). AmpliTaq Gold 360 Master Mix (Life Technologies) and gene specific primer were used for amplification of target genes. The list of primers can be found as Supplementary Table S1 online. The *Gapdh* gene was used as an internal standard. To assess *Tal2* expression in normal mouse embryogenesis, MTC Multiple Tissue cDNA Panels (TAKARA BIO, Japan) were used as templates.

Real-time PCR was performed as described previously^27^. In brief, High Capacity cDNA Reverse Transcription Kit (Life Technologies) was used to synthesize cDNA from 0.5 μg of total RNA. SYBR Green PCR Master Mix (Life Technologies) and 7500 Real-Time PCR system (Life Technologies) were used according to the manufacturer's protocols to perform real-time PCR. The list of primers can be found as Supplementary Table S2 online. The *hydroxymethylbilane synthase* (*Hmbs*) gene was used as an internal standard, and *Tal2* expression levels were normalized to *Hmbs* expression levels. Although we used *Hmbs* as a reference gene in real-time PCR, we have confirmed the constant expression of *Hmbs* in P19 cells after addition of atRA in suspension culture as well as *Gapdh*. Fold expression was defined as fold increase relative to 0 h.

### Microscopy

GFP-fused *Tal2* subcloned into pCAGGS vector^38^. Lipofectamine 2000 (Life Technologies) was used according to the manufacturer's instructions to transfect with the constructed vector into P19 cells. After the transfection, cells were washed with phosphate-buffered saline (PBS), and fixed via incubation with 1% glutaraldehyde (nacalai tesque, Japan) in PBS for 30 min. After washed with PBS, the cells were stained with Hoechst33258 for 30 min. After washed with PBS, the cells were visualized using BZ-8000 (KEYENCE, Japan). Morphological changes of P19 cells were assessed at 48 h after atRA, DMSO, or four agonists treatment. A relief contrast microscope (OLYMPUS, Japan) was used to examine these cells.

### RNA interference

We used pSilencer 3.1-H1 (Life Technologies) as an shRNA vector. The sequences of the shRNA duplexes used RARα knockdown were as follows: *RARα* gene, sense 5′- GCAAGTACACTACGAACAACATTCAAGAGATGTTGTTCGTAGTGTACTTGCTTTTTTGGAA-3′ and antisense 5′- TTCCAAAAAAGCAAGTACACTACGAACAACATCTCTTGAATGTTGTTCGTAGTGTACTTGC-3′, no-target as a control, sense 5′-GTACTATTCGACACGCGAAGTTCAAGAGACTTCGCGTGTCGAATAGTACTTTTTTGGAA-3′ and antisense 5′-TTCCAAAAAAGTACTATTCGACACGCGAAGTCTCTTGAACTTCGCGTGTCGAATAGTAC-3′. Lipofectamine 2000 was used to transfect with each of these constructs into P19 cells. Western blotting probed with an anti-RARα antibody (Santa Cruz Biotechnology, CA) and anti-Beta-Actin antibody (Thermo SCIENTIFIC, CA) were performed to assess RARα knockdown. The influence of *Tal2* expression by RARα suppression was examined by real-time PCR.

## Author Contributions

T.K., R.K., K.K. and H.M. conceived the project. T.K., R.K. and H.M. designed the experiments. T.K., R.K. and K.I. performed the experiments. T.K., S.T. and H.M. wrote the manuscript with discussion of all other authors. All authors reviewed the manuscript.

## Supplementary Material

Supplementary Information *Tal2* expression is induced by all-*trans* retinoic acid in P19 cells prior to acquisition of neural fate
