# Supplementary material for: Tal2 expression is induced by all-trans retinoic acid in P19 cells prior to acquisition of neural fate
Source: Sci Rep. 2014 May 12;4:4935. doi: 10.1038/srep04935 (PMC4017210; doi:10.1038/srep04935)
Supplement: Supplementary Information — Tal2 expression is induced by all-trans retinoic acid in P19 cells prior to acquisition of neural fate [file srep04935-s1.pdf]

Supplementary Information

***Tal2* expression is induced by all-*trans* retinoic acid in P19 cells prior to acquisition of neural fate**

Takanobu Kobayashi [a], Rie Komori [a], Kiyoshi Ishida [a], Katsuhito Kino [a], Sei-ichi Tanuma [b], Hiroshi Miyazawa [a,]*

[a] Kagawa School of Pharmaceutical Sciences, Tokushima Bunri University, 1314-1 Shido, Sanuki, Kagawa 769-2193, Japan
[b] Department of Biochemistry, Faculty of Pharmaceutical Sciences, Tokyo University of Science, 2641 Yamazaki, Noda, Chiba 278-8510, Japan

*Corresponding Author address: Laboratory of Molecular Biology, Kagawa School of Pharmaceutical Sciences, Tokushima Bunri University, 1314-1 Shido, Sanuki, Kagawa 769-2193, Japan. Phone number: +81-87-894-5111. Fax number: +81-87-894-0181.
E-mail address: miyazawah@kph.bunri-u.ac.jp (H. Miyazawa),

Supplementary Figures

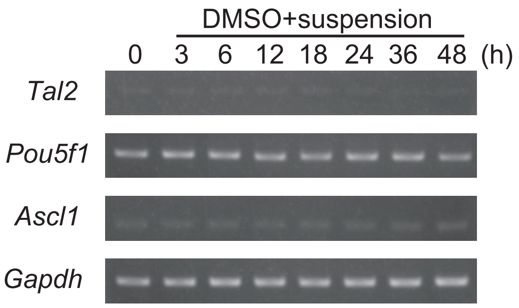

Supplementary Figure S1: Expression of *Tal2*, *Pou5f1*, and *Ascl1* was not altered by DMSO treatment in suspension culture of P19 cells.

P19 cells were treated with DMSO in suspension culture for 0, 3, 6, 12, 18, 24, 36 and 48 h. RT-PCR was performed to examine *Tal2*, *Pou5f1*, *Ascl1*, and *Gapdh* expression.

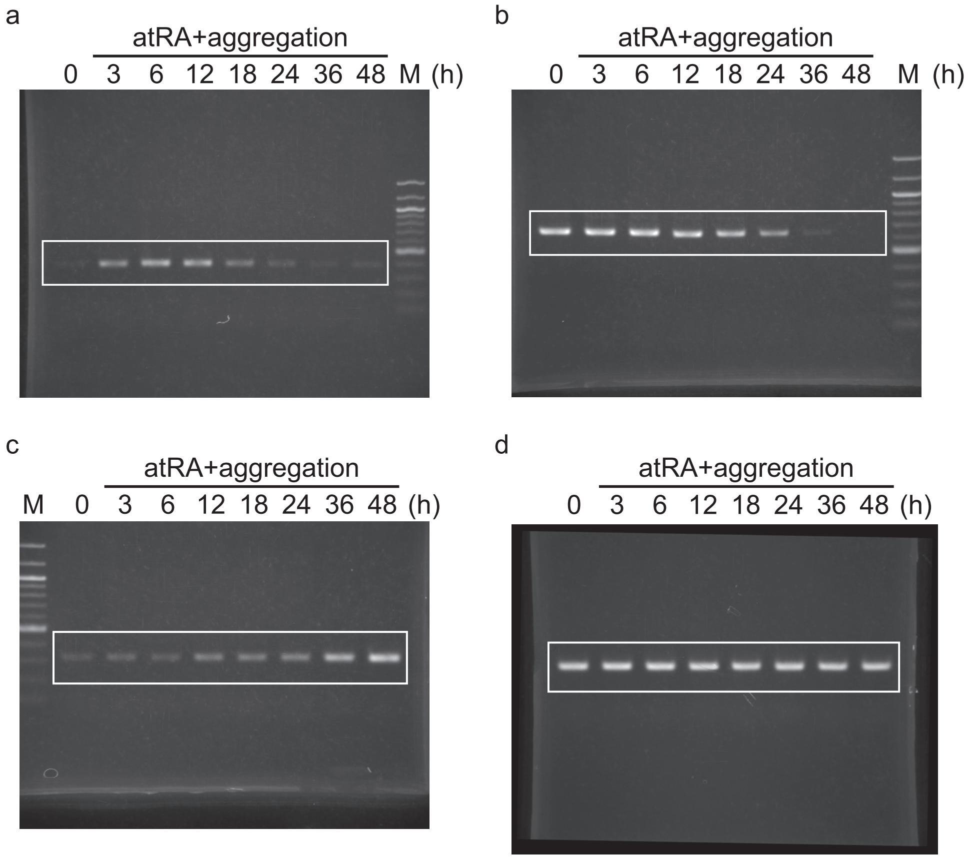

Supplementary Figure S2: Uncropped images of Figure 1a.

P19 cells were treated with atRA in suspension culture for 0, 3, 6, 12, 18, 24, 36 and 48 h. RT-PCR was performed to examine (a) *Tal2*, (b) *Pou5f1*, (c) *Ascl1*, and (d) *Gapdh* expression. We used 100 bp DNA Ladder (New England Biorabs Japan, Japan) as a marker (M). Cropping lines shown as white are indicated in these images.

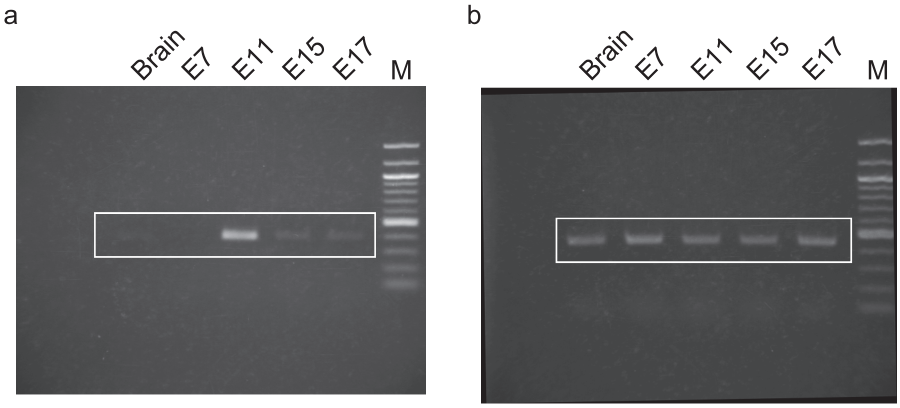

Supplementary Figure S3: Uncropped images of Figure 1c.
RT-PCR was performed to examine (a) *Tal2* and (b) *Gapdh* expression in MTC Multiple Tissue cDNA Panels (E7, E11, E15, E17 and adult brain). We used 100 bp DNA Ladder as a marker (M). Cropping lines shown as white are indicated in these images.

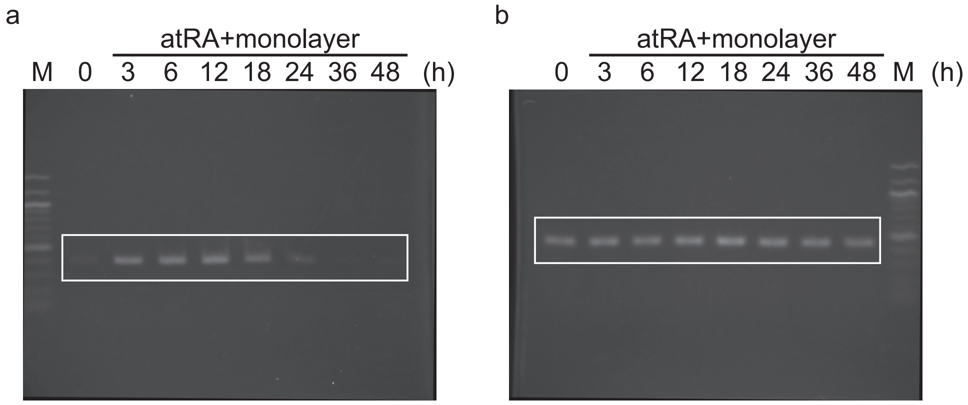

Supplementary Figure S4: Uncropped images of Figure 3a.
P19 cells were treated with 1 μM atRA and cultured in adherent conditions for 0, 3, 6, 12, 18, 24, 36 and 48 h. RT-PCR was performed to examine (a) *Tal2* and (b) *Gapdh* expression. We used 100 bp DNA Ladder as a marker (M). Cropping lines shown as white are indicated in these images.

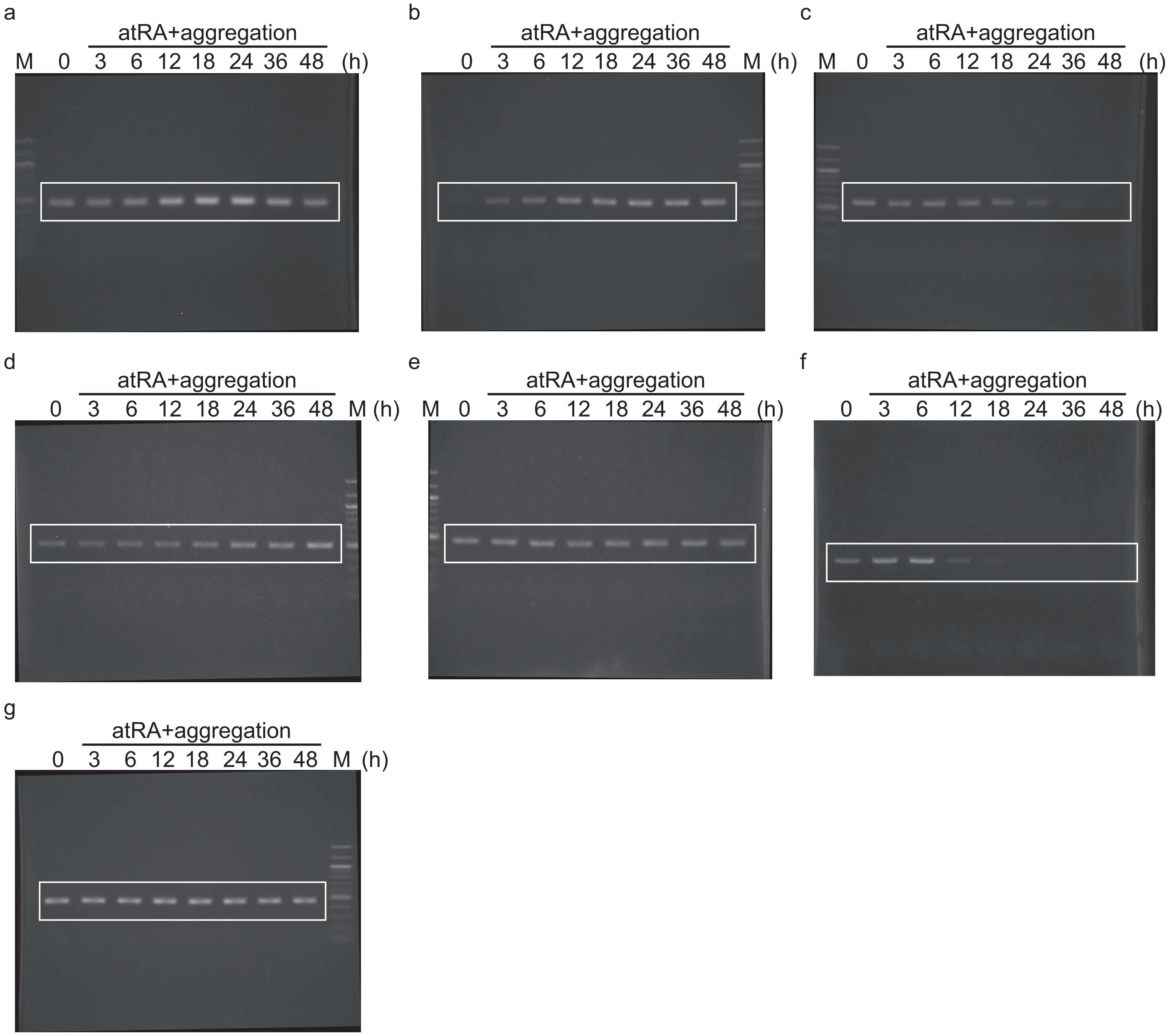

Supplementary Figure S5: Uncropped images of Figure 3b.

P19 cells were treated with atRA in suspension culture for 0, 3, 6, 12, 18, 24, 36 and 48 h. RT-PCR was performed to examine (a) *Rara*, (b) *Rarb*, (c) *Rarg*, (d) *Rxra*, (e) *Rxrb*, (f) *Rxrg* and (g) *Gapdh* expression. We used 100 bp DNA Ladder as a marker (M). Cropping lines shown as white are indicated in these images.

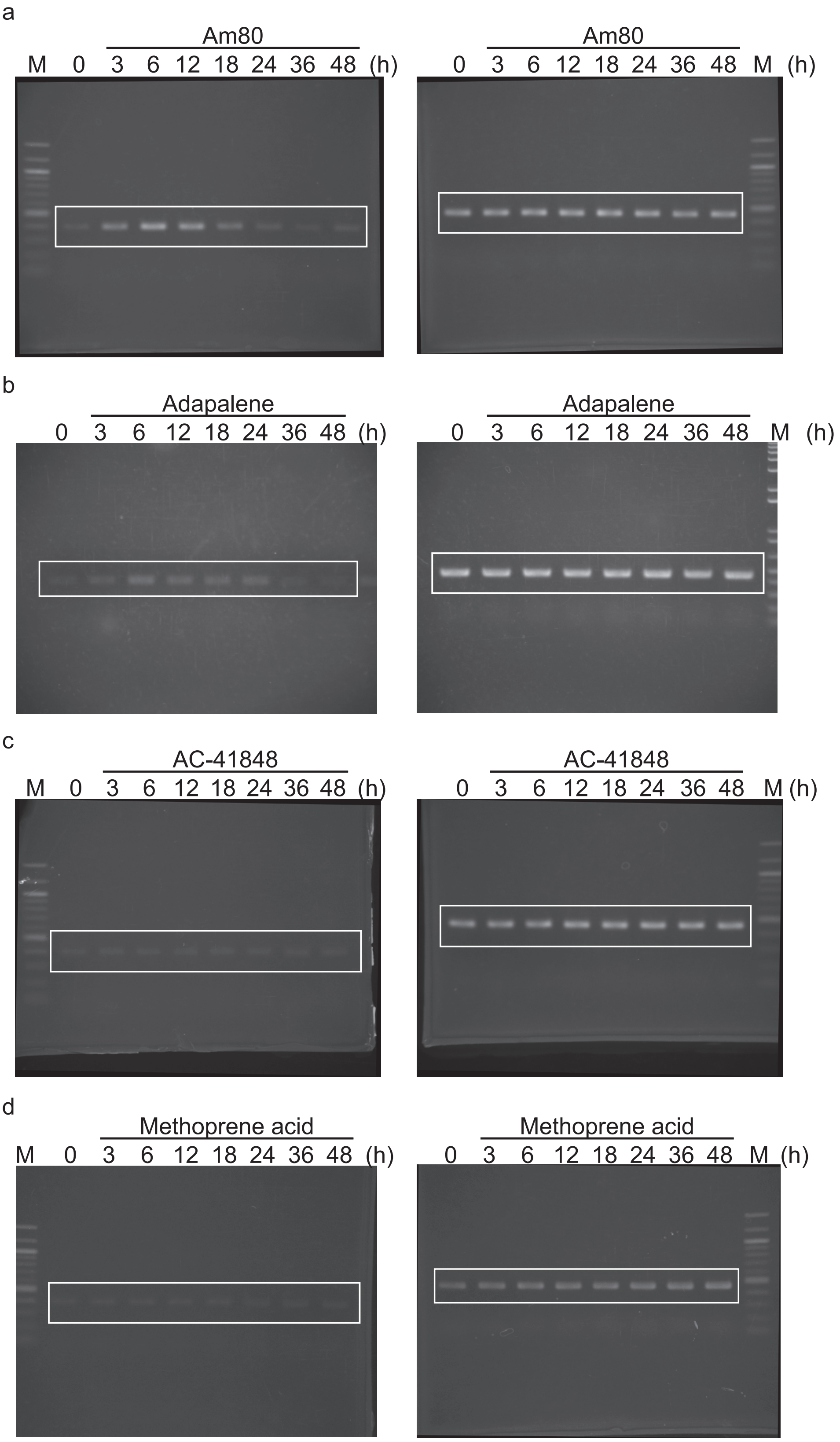

Supplementary Figure S6: Uncropped images of Figure 3c.

P19 cells were treated with four agonists, (a) Am80, (b) adapalene, (c) AC-41848 and (d) methoprene acid in suspension culture for 0, 3, 6, 12, 18, 24, 36 and 48 h. RT-PCR was performed to examine *Tal2* (left) and *Gapdh* (right) expression. We used 100 bp DNA Ladder as a marker (M). Cropping lines shown as white are indicated in these images.

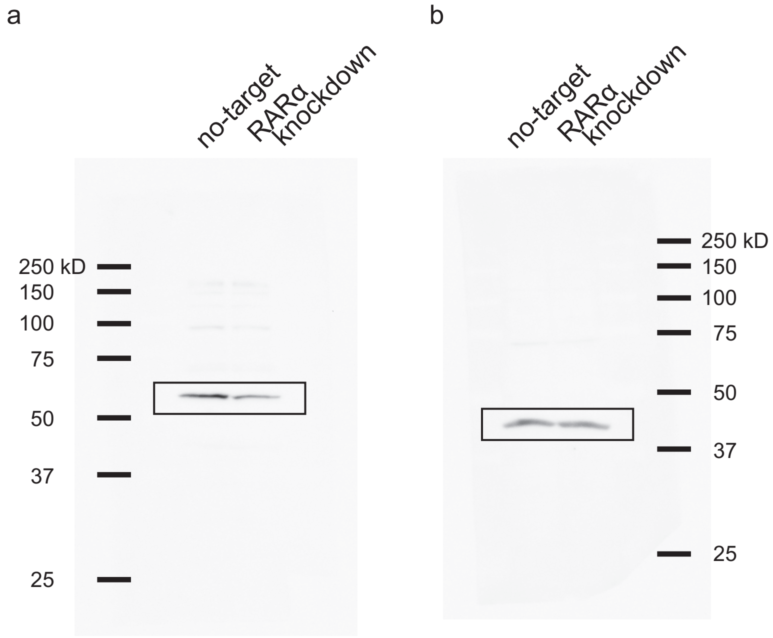

Supplementary Figure S7: Uncropped images of Figure 4a.

Western blotting probed with an (a) anti-RARα antibody or (b) anti-Beta-Actin antibody was performed to assess RARα knockdown. We used Precision Plus Protein™ All Blue Standards (Bio-Rad Laboratories, CA) as a marker. The black bars in this image indicated molecular weight. Cropping lines shown as black are indicated in this image.

Supplementary Tables

Supplementary Tables S1: The primer sequence for RT-PCR.

Genes	Forward primer	Reverse primer
Tal2	ATTGCTCGAGCCCTATTACCC	GGGAAAGAGCCCCAGAATGT
Pou5f	GTTGGAGAAGGTGGAACCAAC	GGACTGAGTAGAGTGTGGTGA
Ascl1	CAACCGGGTCAAGTTGGTCAA	CCAGTTGGTAAAGTCCAGCAG
Rara	CGACGAAGCATCCAGAAGAAC	CGCAGAATCAGGATATCCAGG
Rarb	AAGCCTGCCTCAGTGGATTCA	GCGCTGGAATTCGTGGTGTAT
Rarg	GGAAGCTGTAAGGAACGATCG	TCCATTCGGTCTCCACAGATG
Rxra	CGACTTCTCTACCCAGGTGAA	CAGGTGTAGGTCAGGTCTTTG
Rarb	CCTCAGATCAACTCCACAGTG	ACTCTTCTGCTCCACAGCAAG
Rarg	AGCAGCTCTGAGGACATCAAG	GAGTAGAATGACCTGGTCCTC
Gapdh	ACCACAGTCCATGCCATCAC	TCCACCACCCTGTTGCTGTA

Supplementary Tables S2: The primer sequence for real-time PCR.

Genes	Forward primer	Reverse primer
Tal2	GCAAAGCCTGCATCAAACAG	GGGAAAGAGCCCCAGAATGT
Hmbs	ACTCTGCTTCGCTGCATTG	AGTTGCCCATCTTTCATCACTG
